# Dynamics of Endocrine Disruptor Exposure in Early Life: A Mother–Infant Hair Biomonitoring Study

**DOI:** 10.3390/toxics14020175

**Published:** 2026-02-16

**Authors:** Aikaterini Kalligiannaki, Stella Baliou, Elena Vakonaki, Eleftherios Panteris, Eleftheria Hatzidaki, Manolis N. Tzatzarakis

**Affiliations:** 1 Laboratory of Toxicology, School of Medicine, University of Crete, 70013 Heraklion, Greece; 2 Department of Neonatology/Neonatal Intensive Care Unit, University Hospital of Heraklion, School of Medicine, University of Crete, 70013 Heraklion, Greece

**Keywords:** endocrine-disrupting chemicals, hair, biomonitoring, bisphenol S, parabens, triclosan, organochlorine compounds, early-life exposure

## Abstract

Background: Fetal and postnatal development appears to be influenced in multiple ways by exposure to endocrine-disrupting chemicals (EDCs). We used hair biomonitoring to assess the burden of selected EDCs—bisphenol S (BPS), parabens (PBs), triclosan (TCS), and organochlorine pollutants—in pregnant women and their children at birth and at ten months of follow-up. Methods: Hair samples were collected from pregnant women in Crete at delivery and from their infants shortly after birth and during follow-up. The assessment of EDCs’ burden was performed using liquid and gas chromatography–mass spectrometry (LC-MS, GC-MS). Results: Pregnant mothers had higher BPS levels than their infants at birth, whereas at 10 months’ follow-up, infants exhibited markedly higher BPS concentrations than both their birth levels and the maternal levels, indicating increasing postnatal exposure. Infants at birth had higher TCS levels than their mothers; these levels then declined at follow-up. In contrast, mothers contained higher levels of MeP, EthP, BenP, and ButP levels than those of infants, either at birth or at ten months’ follow-up. Organochlorine compounds were present at low but measurable levels. Significant pairwise comparisons were observed for some of the EDC analytes, mostly between mothers and their infants and between mothers and infants at follow-up. Conclusions: These findings demonstrate constant, compound-specific, and time-dependent EDC burdens, highlighting the importance of prenatal EDC exposure in infants at birth and at ten months’ follow-up compared to that of mothers.

## 1. Introduction

Endocrine disruptors (EDCs) are considered by the World Health Organization (WHO) as substances administered exogenously that hinder the human endocrine system and lead to adverse complications [1,2]. These compounds, which may be natural or synthetic, are common in modern environments—including food, cosmetics, and everyday consumer products—and exposure often occurs through items such as personal-care products, packaging, plastics, metal food cans, household items, detergents, flame retardants, and thermal receipt papers [3,4,5].

There is growing evidence that humans are constantly in contact with these substances through their surroundings, the diets they consume, the products they use, inhalation, skin contact, and perinatal transfer [6,7,8]. In light of EDCs’ effects on human health, the positive association between increased women’s exposure to EDCs and the onset of disorders in offspring’s cardiovascular, neurodevelopmental, or immune systems has been investigated [9,10,11,12,13,14]. On a molecular level, the mechanism of EDCs involves either activating or inhibiting the action of estrogen, androgen, or thyroid hormone pathways, thereby modifying hormone synthesis or metabolism [14].

Because of their widespread use and potential health risks, several EDC classes are top priorities. An essential category of EDCs is bisphenols (BPs). BPs are frequently found in food containers, thermal-printed receipts, and personal hygiene goods, and they have been positively associated with a broad spectrum of disorders [15,16]. In this instance, pregnancy appears to be a crucial period for fetal exposure to BPs, as they cause immunological hyperresponsiveness, even at exceptionally low concentrations [16,17,18]. Interestingly, newborns’ ineffective UDP-glucuronosyltransferase system, which is necessary for BP detoxification and does not fully mature until around two to three months of age [19,20], makes them even more vulnerable to the adverse effects of BPs [21]. In particular, bisphenol S (BPS) is more stable at high temperatures and more resistant to sunlight than bisphenol A (BPA), causing it to be considered as a “safe” substitute for BPA [22]. Accumulating evidence suggests that BPS may exhibit greater endocrine-disrupting and genotoxic activity [23,24,25].

Additionally, parabens (PBs) which are alkyl esters of p-hydroxybenzoic acid, are often used as preservatives against bacteria in food, drink, medicine, cosmetics, and personal hygiene products [26]. In more detail, methylparaben (MeP), ethylparaben (EthP), propylparaben (ProP), benzylparaben (BenP), and butylparaben (ButP) are commonly used PBs in the products mentioned above. According to biomonitoring studies, most participants have shown measurable levels of PBs in their urine due to exposure through ingestion, inhalation, and skin contact [27,28]. Interestingly, PBs pose a considerable risk to human health [29]. In particular, PBs can negatively be associated with malignancies like breast cancer, metabolic disorders such as obesity, neurodevelopmental complications, reproductive system problems, and genotoxicity [30,31,32,33,34]. In addition, children and pregnant women have shown to harbor higher levels of PB exposure than adults [29,35,36]. According to these lines of research, BPs and PBs can develop into serious health issues [29].

Triclosan (TCS), an antimicrobial agent, is prevalent in a range of personal care and household products [37]. Regarding the adverse effects of TCS, it has been shown that exposure to TCS is associated with neurodevelopmental and reproductive toxicity, underscoring the need for the implementation of regulatory measures [16].

In addition, persistent organic pollutants (POPs) such as hexachlorobenzene (HCB) and the organochlorine DDT and its main metabolites [op dichlorodiphenyldichloroethylene (opDDE) and pp dichlorodiphenyldichloroethylene (ppDDE), along with op-dichlorodiphenyldichloroethane (opDDD) and pp-dichlorodiphenyldichloroethane (ppDDD)] remain an issue due to their persistence in the environment long after their use has been banned. These compounds are associated with carcinogenesis, endocrine dysfunction, and neurotoxicity [38,39].

In the field of EDC exposure during pregnancy and infancy, multiple retrospective studies have elucidated the impact of EDCs on pregnant women’s urine [40,41,42,43], considering that PBs have a half-life of less than 24 h and are rapidly metabolized in urine [44]. In this context, a strong association of MeP, BenP, ButP, EthP, and TCS was revealed in the urine and amniotic fluid of pregnant women in a non-significant manner [45]. In particular, MeP was identified as the prevalent compound in the amniotic fluid of pregnant women, whereas TCS was found to prevail in the urine samples [45]. Interestingly, MeP levels in amniotic fluid were negatively associated with maternal age, whereas maternal body mass index (BMI) was positively associated with both urine and amniotic TCS levels [45]. In addition, PBs and TCS levels did not show any statistically significant correlations with newborn somatometric traits or health status [45].

To the best of our knowledge, this is the first study to apply hair biomonitoring to simultaneously assess PBs, BPS, TCS, and organochlorine compounds in paired maternal–infant hair samples, thereby enabling evaluation of long-term, cumulative exposure dynamics. Through hair biomonitoring, our findings provide important information about EDC concentrations in infants at birth and ten-month follow-up that are crucial because neonates and infants are thought to be particularly vulnerable to EDC exposure [46,47], as they have limited detoxification and excretion capacities and are more susceptible to hormonal deficiencies [48]. This study underscores the significance of prenatal exposure to EDCs during critical developmental windows.

Based on our previous findings, the novelty of this study lies in hair biomonitoring to assess long-term and cumulative exposure of mothers and their infants to EDCs at birth and during follow-up [49,50,51].

## 2. Materials and Methods

### 2.1. Hair Sampling

All participants were citizens of the island of Crete, Greece, and the hair samplings were performed at the Department of Neonatology at the University Hospital of Heraklion. Before collecting samples and completing questionnaires, parents of the neonates received study details and gave written consent to participate. Mothers’ personal information, including age, somatometric traits, educational level, employment status, and residential area, was collected via questionnaires. Additionally, information about pregnancy was also collected, including gestational age, maternal and infant health problems, and somatometric characteristics of the newborns (birth weight, length, and head circumference).

Maternal hair was collected from the back of the head, close to the scalp; if the total length exceeded 9 cm, the first 9 cm (0–9 cm) was chosen for analysis. Given the accepted mean growth rate of head hair of 1 cm per month, a 9 cm hair segment represents the last 9 months of exposure (pregnancy period). The whole hair length (0.5 to 5 cm) of the newborns was collected and analyzed in the study. Following collection, the samples were covered with aluminum foil and stored at room temperature in a dry, dark location until analysis. The Ethics Committees of the General Hospital of Heraklion (43(1496)/27-12-2018) and the University of Crete (42/22.11.2018) both authorized the study. Specifically, hair samples from mothers and their newborns were collected at the neonatal intensive care unit (NICU) of the General Hospital of Heraklion using steel scissors, and hair samples from infants at the 10-month follow-up were collected at their homes from 2019 to 2021. Immediately after collection, hair samples were wrapped in aluminum foil and stored in the dark at room temperature until further analysis. The current population comprises mother–infant pairs. Among these participants, there were initially 9 pairs of twins (i.e., 9 mothers who each gave birth to twins, resulting in 18 twin infants). In the analytical dataset, mothers with twins are represented more than once, as each mother–infant dyad—including both twins—is treated as a distinct analytical unit to accurately capture individual exposure profiles. A repeat hair sampling was conducted in the infants at the age of 10 ± 1 months (follow-up phase), where 4 pairs of twins (8 infants) were available for repeat sampling. Due to the small number of twins present at follow-up, no separate statistical analysis was performed for this subgroup.

In this study, 59 women responded to the questionnaires, of whom 47 women consented to provide hair for analysis. The somatometric characteristics were derived from 68 newborns, including 9 twins. In total, 47 head hair samples from the mothers, 64 hair samples from infants at birth, and 24 hair samples from infants at follow-up were provided as the available hair quantity for analysis and included in this study. Interestingly, the study cohort included both term and preterm neonates (the vast majority), as recruitment was conducted at the neonatal intensive care unit (NICU) of the General Hospital of Heraklion. As a result, a proportion of newborns were born preterm with low birth weight.

For the assessment of analyte exposure, pairwise comparisons were performed for each analyte across the following groups:

- Mother and infant at birth: pairwise comparison between the mother and her infant immediately after delivery;
- Mother and infant at follow-up sampling time: pairwise comparison between the mother and her infant at the follow-up;
- Infant at birth and at follow-up sampling time: pairwise comparison within the same infant, comparing concentrations at birth and at follow-up.

### 2.2. Chemicals and Reagents

BPS, MeP, ProP, EthP, BenP, ButP, TCS, and ammonium acetate (≥ 98%) were purchased from Sigma-Aldrich (St. Louis, MO, USA). Methanol and acetonitrile (LC-MS grade) were obtained from Fischer Chemicals, Loughborough, UK. Phenobarbital, which was used as an internal standard (IS), was purchased from Lipomed AG, Arlesheim, Switzerland. The HCB, DDEs, and DDDs, as well as the 1,2,3,4-tetrachloronaphthalene (TCN) used as an external standard, were obtained from Dr. Ehrenstorfer Laboratories (Augsburg, Germany). Sodium hydroxide and hexane were purchased from Merck, Darmstadt, Germany, and dichloromethane from Fisher Chemical, Longborough, UK. Nanopure water was obtained from Direct-Q 3UV water purification system (Merck, Darmstadt, Germany).

### 2.3. Extraction of PBs, BPS, and TCS from Hair

The washing process was common for all hair samples. Firstly, the hair samples were washed with water (twice), methanol (twice), and hexane (twice), and then dried at 50 °C to avoid any external contamination from the hair matrix. After drying, the hair was cut into small pieces (mm), weighed, and placed in glass vials (6 mL). The extraction protocols for PBs, BPS, and TCS have been previously described [49,50,51,52,53]. In particular, 50 ng of IS and 2 mL of LC-MS grade methanol were added to each sample, and the vials were placed in an ultrasonic bath for 2 h. The extraction process was carried out once again. The methanol extracts were separated, mixed, and dried under a nitrogen stream at 35 °C. In the dry residue, 100 μL of methanol was added, and 10 μL was used for analysis by liquid chromatography–mass spectrometry (LC-MS).

### 2.4. Extraction of Organochlorine Compounds from Hair

After completing the analysis of PBs, TCS, and BPS, 75 μL of the remaining solution was added back to the hair samples, and the extraction of organochlorines was carried out as follows.

Hair samples were treated with 2 mL NaOH 5N in an ultrasonic bath for 1 h. The extraction was performed mechanically with 2×3 mL hexane-dichloromethane (3:1) for 10 min [51]. The organic layers were combined, 10 ng of TCN was added, and the solution was evaporated to dryness under nitrogen at 35 °C. The dry residue was reconstituted in 200 μL hexane and transferred to solid-phase microextraction (SPME) vials. When hexane was evaporated to dryness, 2 mL water and 0.6 g NaCl were added, and the SPME vials were sealed with PTFE/silicon septum caps [54]. A 65 μm PDMS/DVB type fiber (Supelco, Bellefonte, PA, USA) was used for headspace online extraction, and it was agitated at 250 rpm for 20 min at 90 °C. The fiber tip was placed in the GC-MS’s injection port once the adsorption procedure was complete, and it was maintained there for three minutes until the analytes were completely released.

### 2.5. Instrumental Analysis

#### 2.5.1. Liquid Chromatography-Mass Spectrometry

A Shimadzu LC-MS-2010EV (Kyoto, Japan) was used for the detection and quantification of PBs, TCS, and BPS, and a C18 column (Supelco) (250 mm, 4.6 mm, 5 μm) was used for the separation of the analytes. The mobile phase that was used for the elution of the compounds from the chromatographic column consisted of 5 mM ammonium acetate (solvent A) and acetonitrile (solvent B). Gradient elution was initiated with a concentration of 15% of solvent B (time: 0–1 min), followed by 95% solvent B (time: 1–20 min), remained stable at 95% (time: 20–24 min), and finally returned to 15% (time: 24–29 min). The flow rate of the mobile phase was 0.6 mL/min, and the oven temperature was 30 °C. The monitoring of the analytes was performed with a single quadrupole mass filter combined with atmospheric pressure chemical ionization (APCI) in the negative mode. The interface, CDL, and heat block temperatures were set up at 400 °C, 200 °C, and 200 °C, respectively. The nebulizing gas flow rate was 2.5 L/min.

#### 2.5.2. Gas Chromatography–Mass Spectrometry

The instrumental analysis of organochlorine pollutants (HCB and DDT isomers) was performed using gas chromatography–mass spectrometry (Shimadzu, QP-2010, Kyoto, Japan), and the separation was conducted on an SLBtm-5ms (Supelco) capillary column (30 m × 0.25 mm × 0.25 μm). The oven temperature was initially set at 60 °C and gradually raised to 180 °C at a rate of 15 °C/min, then set at 250 °C with an increasing rate of 4 °C/min, and the final temperature was set at 300 °C. Pure helium (99.999%) was used as a gas carrier with a flow rate of 1 mL/min. The MS interface and ion source temperature were set at 310 °C and 230 °C, respectively.

### 2.6. Statistical Analysis

All statistical analyses were performed using IBM SPSS Statistics, version 26 (IBM Corp., Armonk, NY, USA), and all data visualizations were conducted in Python (version 3.10). Data cleaning and management were undertaken using the pandas library (version 1.5.3). Data were expressed as mean ± SD, as median (IQR), or as numbers and percentage *n* (%) where appropriate.

For each analyte, box plots were constructed to illustrate the distribution across three groups: (1) mothers, (2) infants at birth, and (3) infants at follow-up. The normality of the data was assessed using the Shapiro–Wilk test. As all variables deviated from a normal distribution, non-parametric statistical tests were employed throughout. Specifically, for each pairwise comparison (mother vs. infant at birth, mother vs. infant at follow-up, and infant at birth vs. infant at follow-up), the Wilcoxon signed-rank test was applied. For comparisons involving more than two groups, the Kruskal–Wallis test was utilized to assess differences across feeding modalities. All statistical tests were two-tailed, and a *p*-value < 0.05 was considered statistically significant.

Visualisations were generated using matplotlib (version 3.6.3) with the seaborn “whitegrid” style for enhanced clarity and consistency. The analysis also employed numpy (version 1.24.0) for numerical operations and scipy (version 1.9.3) for statistical testing. For each analyte, box plots depicted the median, interquartile range (IQR), and outliers; means were also indicated where relevant. Numeric variables containing missing or non-numeric values were handled by coercion to NaN and subsequent exclusion from the analysis.

## 3. Results

### 3.1. Method Validation

The parameters of linearity, limits of detection (LOD), limits of quantification (LOQ), recovery, accuracy, and precision (%RSD) were assessed to examine the performance of the analytical methods. The limits of detection (LOD) and limits of quantification (LOQ) were determined using signal-to-noise (S/N) criteria: S/N = 3 for LOD, and S/N = 10 for LOQ. The retention times (min), the monitoring ions for each compound, and the results from the method validation are presented in Table 1 for all analytes. The target ion was used for the quantitative determination of the analytes, whereas the Q1 ion was employed for qualitative confirmation.

Multi-standard solutions of BPS, parabens, and TCS were prepared in methanol at levels of 0, 25, 50, 100, 250, and 500 ng/mL, while for HCB and DDT isomers the levels were 0, 0.25, 0.5, 1, 2, and 5 ng. Standard solutions were used to generate the calibration curves, and R^2^ correlation coefficients assessed the instrument’s linearity for each compound. For BPS, TCS, and parabens, the correlation coefficients exceeded 0.99, and for organochlorine compounds, the coefficient was above 0.98 (Table 1).

Hair samples were used to prepare spiked samples at levels of 0, 5, 10, 25, 50, 100, 500, and 1000 pg/mg for BPS, BenP, ButP, and EthP, as well as levels up to 2500 pg/mg for MeP, ProP, and TCS. Similarly, spiked hair samples were prepared at levels of 0, 2.5, 5, 10, 25, and 50 pg/mg for HCB and DDT isomers. These spiked samples were processed using the same protocol as authentic hair samples, and their calibration curves were used to determine the concentrations in the authentic samples and evaluate the method. For each batch, at least one standard multi-component mixture of substances, two spiked samples with known concentrations, and blank solvent samples were analyzed in order to ensure the proper operation of the instrument. The method’s linearity was assessed using the relation coefficients R^2^ from the spiked calibration curves, which were 0.9901 for BPS, 0.9972 for TCS, and 0.9812 for ProP to 0.9937 for MeP. For organochlorine compounds the method linearity ranged from 0.9924 (ppDDE) to 0.9981 (HCB) (Table 1).

The average recovery of the method was 92.8% for BPS and 102.7% for TCS, and for parabens it ranged from 81.9% (ButP) to 102.2% (EthP). For organochlorine compounds, recovery ranged from 72.8% for HCB to 85.4% for ppDDE (Table 1). The precision of the method was expressed as the percentage relative standard deviation (%RSD) which was 12.4% for BPS and 18.6% for TCS, and for parabens it ranged from 15.5% (ProP) to 20.4% (MeP); it was 18.2% for HCB and ranged from 16.0% to 17.4% for DDT isomers (Table 1). The mean percentage accuracy values ranged from 97.2% to 103.1% for HCB and DDT isomers and from 84.5% to 122.4% for BPS, TCS, and parabens (Table 1). The achieved LOQs were 1.9 pg/mg for BPS, 2.5 pg/mg for TCS, and from 1.0 to 2.6 pg/mg for parabens. For organochlorines, LOQs were 0.27 pg/mg for HCB and ranged from 0.21 pg/mg to 0.26 pg/mg for DDT isomers (Table 1).

### 3.2. Demographics

Table 2 summarizes the demographic and anthropometric characteristics of the participating mothers, categorized by area of residence (urban vs. rural). Variables such as maternal age, height, pre-pregnancy weight, weight at delivery, and the corresponding changes in weight (ΔWeight) and body mass index (ΔBMI) during pregnancy are included to report the demographic and anthropometric characteristics of the participating mothers. In addition, educational level and employment status are needed to show the socioeconomic status of the study population. The mean age of the mothers was 33.1 years. The mean BMI values before pregnancy and at gestation week were 24.86 and 28.52 kg/m^2^, respectively. Specifically, the mean gestational weight gain (ΔWeight) was 10.25± 5.35 kg, and the mean gestation week was 35.03 ± 3.46. The majority of the women had a university degree (50.8%), and most were employed in the private sector (66.1%).

In Table 3, demographic data and health issues of newborns are presented by living area. There was no significant difference in the somatometric measurements of newborns between urban and rural areas (*p* > 0.05). The mean ± SD weight of all newborns was 2292 ± 593 g, the average height was 46 ± 4 cm, and the average head circumference (HC) was 32 ± 2 cm. Most of the newborns were boys (67.1%), and the most common health issues observed during the first six months after birth were respiratory disorders (17.1%).

### 3.3. Maternal Environmental Exposure and Use of Cosmetic Products

The most frequently cited sources of environmental exposure were bottled water (regularly consumed by 61.0% of mothers) and tap water from the public network (27.1%). Domestic roof-top water tanks and plastic plumbing were also common, reported by 40.7% and 35.6% of participants, respectively.

During pregnancy, the overall use of many personal-care products decreased (Table 4). Daily body deodorant use dropped from 79.7% to 57.6% (*p* < 0.001). Daily perfume use decreased from 55.9% to 28.8% (*p* < 0.001), and makeup application lessened from 47.5% to 23.7% (*p* = 0.007). Nail polish use shifted toward non-use, with “never” responses rising from 23.7% to 59.3% (*p* < 0.001). Frequencies of hair spray/gel and body lotion use also declined (*p* = 0.004 and *p* = 0.003, respectively), while sunscreen use declined across daily and weekly categories (*p* = 0.019). Hair dye frequency showed a downward trend that did not reach statistical significance (*p* = 0.066). In contrast, shower gel use remained at similar levels, with about two-thirds of women reporting daily use both before and during pregnancy (*p* < 0.001), due to minor shifts within less-common categories.

In none of these products was the frequency of use increased, nor was the usage shifted to less frequent intervals, such as from daily to weekly. Fortunately, even though 17 women were frequent smokers for an average of 8.5 years, only 3 of them reported rare smoking during their pregnancy. Eleven women stated that they were occasional smokers and did not smoke throughout their pregnancy. Classification of the sample into smokers and non-smokers did not reveal any connection between smoking and ED concentrations.

### 3.4. Biomonitoring Data

Table 5 summarizes the detection frequency (%), mean ± SD, and concentration range (pg/mg) for each analyte in mothers, infants at birth, and infants at follow-up. BPS, MeP, ProP, and TCS are universally present in mothers (100.0%) and remain highly prevalent among infants at birth (87.1–100.0%) and at follow-up (87–95.8%). EthP is detected in 92.6% of mothers but only 38.7% of infants at birth, increasing to 70.8% at follow-up. BenP and ButP have lower frequencies across all time points, with detection ranging from 17.7% to 61.1%.

HCB shows high and relatively stable detection rates (76.0% in mothers, 75.0% in infants at birth, and 63.6% in infants at follow-up). ppDDE also shows a consistently high positivity: 90.0% in mothers, 88.3% in infants at birth, and 77.3% at follow-up. opDDE ranges from 44.0% in mothers to 73.3% and 72.7% in infants at birth and follow-up, respectively. In contrast, opDDD remains low across all groups (≤31.7%), with detection in mothers being very rare (8%) (Table 5).

Mean concentrations of several EDCs were highest in mothers, while in infants, postnatal increases were observed for BPS (from 79.24 ± 251.21 to 289.2 ± 250.98 pg/mg), ProP (from 460.62 ± 589.22 to 1003.41 ± 2292.79 pg/mg), and ButP (from 6.64 ± 19.92 to 21.19 ± 46.24 pg/mg). Organochlorine residues—including HCB, pp and opDDE and opDDE—remained at low exposure levels (mean < 15 pg/mg) (Table 5).

### 3.5. Feeding Methods Comparison

We proceeded with measuring EDCs in infants at the ten-month follow-up, based on their feeding type. Our results showed no association in analyte concentrations at follow-up (Table 6). To prevent baseline bias from affecting these comparisons, analyte levels were also examined at birth. This analysis revealed that exposure levels were similar between feeding groups at baseline. No statistically significant differences were found between feeding groups for any analytes. Although median concentrations for some analytes were numerically higher in breastfed or mixed-fed infants, none of these differences were statistically significant. For example, MeP concentrations were higher in breastfed (953.15 pg/mg, IQR: 119.11–4812.35) than in mixed-fed infants (414.67 pg/mg, IQR: 229.73–1347.93), but this difference was not statistically significant (*p* = 0.920). Notably, median TCS levels were also higher in breastfed infants, but this difference did not reach significance. Overall, these findings suggest that the feeding type does not influence exposure to these analytes during infancy.

### 3.6. Sex Comparison

Sex-based comparisons of analyte concentrations at birth and at follow-up are shown in Table 7. At birth, the distributions of all measured analytes were similar between boys and girls, with no statistically significant differences observed for any analyte (all Mann–Whitney U test *p*-values above 0.05). For example, the median BPS concentration was 18.78 pg/mg (IQR: 13.25–44.47) in boys and 23.86 pg/mg (IQR: 16.90–47.92) in girls (*p* = 0.290). Similar patterns were seen for MeP, EthP, ProP, and other analytes, indicating that, in this study, infant sex did not significantly correlate to exposure.

At follow-up, as presented in Table 7, the absence of sex-based differences persisted. Median and interquartile range values for all analytes remained comparable between male and female infants, with *p*-values consistently exceeding the significance threshold.

### 3.7. Pairwise Comparison for Mothers and Infants

**Figure 1:**
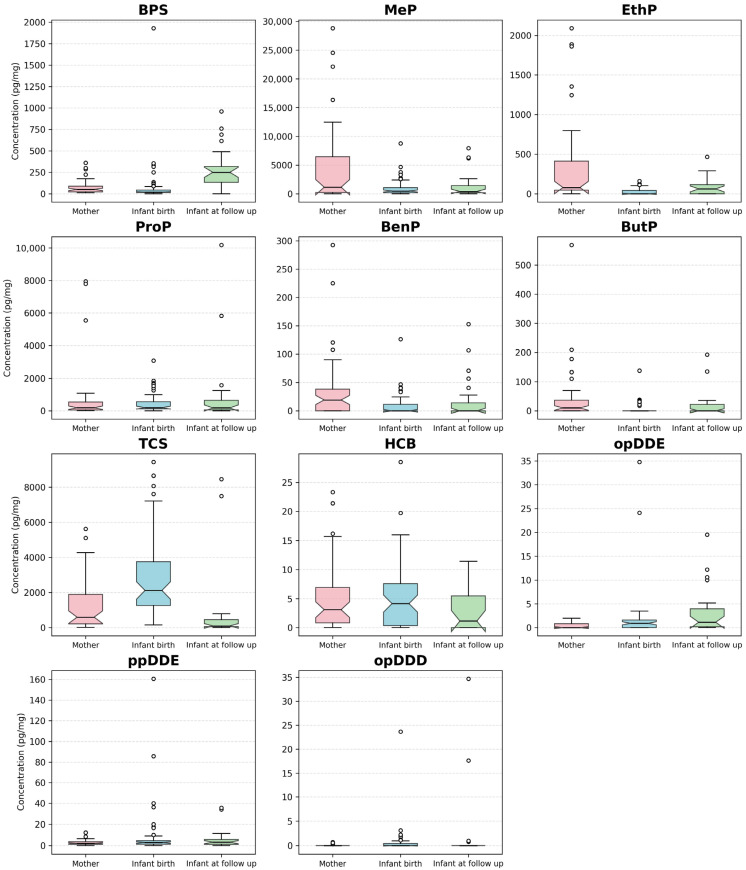
Median, IQR, and analyte concentrations (pg/mg) for mothers and infants at birth and at follow-up. IQR: interquartile range.

Pairwise comparisons of maternal and infant analyte concentrations, both at birth and at follow-up, are summarized in Table 8 and Figure 1. In most EDC cases, time-specific differences appeared, while others remained broadly comparable across the dyad. The Wilcoxon signed-rank test identified several statistically significant differences between mothers and infants and within infants across the two sampling points. For BPS, maternal levels were higher than infant levels at birth, whereas by ten months, infants had markedly higher levels than those of their mothers. Significant pairwise differences were observed between mothers and their infants (*p* = 0.001), between mothers and infants at follow-up (*p* < 0.0001), and between infants at birth and follow-up (*p* = 0.001).

Among PBs, MeP, EthP, ButP, and BenP showed the highest mean levels in pregnant women, with infant concentrations being lower at birth and increasing by ten months. For these compounds, significant pairwise differences were observed between the levels detected levels in mothers’ hair and the hair of the infants at birth (*p* < 0.05) (Table 8). No significant pairwise differences were observed for ProP between mothers and their infants at birth and at follow-up nor between infants at different sampling time points (*p* > 0.05).

TCS showed the most precise pattern: newborns had much higher TCS levels than their mothers at birth, but infant concentrations had decreased at ten months. Significant pairwise differences were observed for TCS between mothers and infants at birth (*p* < 0.0001), between mothers and infants at follow-up (*p* = 0.014), and between infants at different sampling time points (*p* = 0.001).

Organochlorine compounds were less common but still detectable across all groups. Significant pairwise differences were found for opDDE, between mothers and infants at birth and at follow-up. For opDDD, significant differences were only seen between mothers and infants at birth. No significant correlations were revealed between pairs for HCB and ppDDE.

## 4. Discussion

To the best of our knowledge, this is the first study to use hair biomonitoring to longitudinally assess maternal and infant exposure to EDCs, providing new insights into fetal and postnatal exposure dynamics. The present study was designed to characterize EDC exposure only, providing initial evidence of EDC patterns and dynamics during early life, without further evaluation of clinical, endocrine, or health outcomes. In particular, our study first offers one of the most comprehensive and novel assessments of early-life exposure to various classes of endocrine-disrupting chemicals (EDCs), including BPS, PBs, TCS, and organochlorine compounds (HCB, DDTs), using hair samples from mothers and their infants at birth and at 10 months. At birth, marked maternal–newborn significant pairwise differences were observed for BPS, MeP, EthP, BenP, ButP, TCS, ppDDE, and opDDD. By the ten-month mark, significant maternal–infant pairwise differences persisted for BPS, EthP, TCS, and opDDE. Within infants, BPS concentrations increased and TCS concentrations decreased between birth and follow-up, reflecting divergent postnatal exposure. However, the dynamics of EDC exposure are not different according to feeding type or infant’s sex.

First, the primary novelty of this study is the use of hair, which provides a more accurate matrix for assessing EDC exposure during pregnancy compared to urine. The hair accumulates chemicals over weeks to months, offering a comprehensive measure of long-term exposure from both systemic circulation and the environment. Thus, hair is a suitable substrate due to its non-invasive collection, stability, and capacity to monitor long-term EDC exposure, which is valuable for assessing maternal and newborn exposure during fetal and neonatal development. In infants, scalp hair has been used as a matrix to assess early-life exposure to environmental contaminants, supporting its utility for evaluating fetal exposure. In particular, a positive relationship between 4,4′-dichlorodiphenyldichloroethylene (pp’-DDE) levels in mothers and infants has been reported [55]. Infant hair has also been used for biomonitoring prenatal exposure to PBs [56,57] and DDTs [58]. Concerning the biomonitoring of PB levels in infants’ hair and urine matrices, an insignificant relationship was reported between the PB concentrations of hair and urine, except for isopropylparaben (i-PropPB) [57]. Interestingly, a higher concentration of propylparaben (PropPB) in boys’ hair was associated with a higher risk of obesity [57].

Regarding infant–mother paired data for urine or blood, it has been shown that the median PB levels in pregnant women’s urine (or blood) and those of their infants tend to be in the range of ng/mL in Korea and the United States [59,60,61]. For instance, the TESIE cohort of children aged 3–6 years old presented with median MenP, ProP, and EthP concentrations of 57 ng/mL, 8.5 ng/mL, and 1.4 ng/mL, respectively, in urine [61]. In contrast, the 2005–2006 National Health and Nutrition Examination Survey (NHANES) found that children aged 6–11-years had mean urinary concentrations of 33.5 ng/mL MenP and 3.41 ng/mL ProP [61]. In a Korean study, median urinary MenP concentrations of 97 ng/mL, ProP of 2.9 ng/mL, and EthP of 4 ng/mL were reported in newborn urine collected within 48 h after delivery [60].

Even though urine biomonitoring has been routinely utilized to measure the levels of EDCs, this work emphasizes the need for hair analysis as a supplementary method to monitor cumulative, long-term exposure to EDCs during pregnancy and infancy, whereas urine represents short-term excretion of metabolized compounds. In particular, our study has highlighted that mean PB concentrations in infants were lower than those in pregnant women, with the exception of ProP, for which ten-month-old infants showed higher levels than those of mothers. In line with the concept of EDC postnatal exposure, the PB levels were lower in newborns and tended to be higher in ten-month-old infants. The concentration differences for most analytes between mothers and infants support previous findings that placental transfer of many EDCs is partial and selective [62,63].

Regarding the sources of PBs, they are present in various cosmetic/personal-care product types (make-up, nail polish, body cream, sunscreen, hair dye, shower gel, hair spray, shampoo, body deodorant, and perfume) [64,65,66,67,68], and the primary routes of exposure to PBs are through dermal contamination [69]. Previous research has highlighted that exposure to BPs can happen through both breastfeeding and skin contact with plastic devices that are frequently used in neonatal intensive care units [11,12,14]. In this context, it has been shown that items offered in neonatal intensive care units (NICUs) included either PBs or BPs [70]. In particular, light therapy glasses, tapes, and creams contained 80% PB frequency, with concentrations measured in the range of ng/mL. In contrast, dummies, gloves, and intravenous (IV) devices contained approximately half the PB frequency and the BPA frequency [70]. In addition, another recent study highlighted that the strongest predictor of urinary paraben biomarker exposure was lotion use within the previous 7 days in infants’ lives, when infants were aged 1–3 months or 12 months [56]. The urinary concentrations of MeP, EthP, and ProP followed the same pattern in infants aged either 1 to 3 months or 12 months, indicating that the levels are due to increased lotion use [56]. Given the short half-life of PBs (less than 24 h), there was no association between PB levels at 1–3 months and those at 12 months. However, MeP and ProP showed statistically significant correlation in the 12-month-old infants [56]. In particular, infants who used lotion had 189% higher concentrations of ProP and 355% higher concentrations of MeP than infants who did not use lotion during the previous seven days [56].

Consistent with our study’s PB results in pregnant women, other researchers have shown that MeP and EthP were detected in nearly all pregnant women, reflecting their widespread use. At the same time, ButP and BenP were detected at much lower concentrations and in fewer occurrences. Ιn pregnant women in the Puerto Rico pregnancy cohort, the urinary PB, TCS, and BP concentrations were shown to be significantly correlated with different personal-care products (soap, sunscreen, lotion, and cosmetics) used over the previous 48 h [71].

In addition to above, we have conducted repeated measurements of BPS concentration to elucidate the burden of BPS during infancy. Our results have highlighted the windows of fetal BPS transfer in newborns and infants over a ten-month follow-up. Interestingly, our study has shown differences in BPS levels between mothers and newborns, between mothers and infants at 10-month follow-up, and between infants at birth and at 10 months. Specifically, the median BPS concentration increased in infants at birth from 19.35 pg/mg to 250.4 pg/mg at 10 months, reaching levels well above those observed in pregnant women (51.08 pg/mg). In our study, the median BPS concentrations in hair of 10-month-old infants (250.40 pg/mg) were consistent with the overall median BP levels reported in the hair of Spanish children aged 4–12 years (311.33 ng/g) [72].

Furthermore, the BPS levels in infants can be determined through infants’ contact with BPS-containing bottle materials, plastic liners, formula packaging, plastic toys, and pacifiers. Even though each of these microexposures is insignificant on its own, frequent contact can cause them to accumulate and significantly increase the body’s total chemical load in infants [73]. To support this, recent research has shown that high mean BPS concentrations in newborns are due to commonly used non-food items in neonatal care, including pacifiers. Specifically, pacifier use was positively associated with both total BPS and unconjugated BPS concentrations in the urine of neonates [22].

Regarding BPS concentrations in pregnant women, the detection rate was 100%, with a mean concentration of 75.42 pg/mg in our study. This likely occurs because BPS can be present as a contaminant or additive in the packaging of personal-care products and cosmetics [74]. In this context, differences in BPS concentration among pregnant women worldwide can be explained by occupation. In a study conducted in Asia, mothers working as cashiers, office workers, instructors, and salespeople all showed high urine BPS levels during pregnancy [75].

In line with the above information, our study has also provided strong evidence that TCS concentrations in hair peak at birth: newborns had substantially higher TCS levels than their mothers, and these levels were higher at birth than at ten months of age. By the 10-month follow-up, infant TCS concentrations had declined and were lower than maternal levels. Interestingly, the peak TCS concentration in hair of newborns probably reflected its bioaccumulation, probably due to limited fetal metabolism and an immature detoxification system, as TCS’s poor glucuronidation and sulfation abilities prevent newborns from effectively metabolizing or excreting it [76].

Last, the bioaccumulative features of the organochlorine compounds in infants are further supported by the detection of hexachlorobenzene (HCB) and DDT metabolites such as opDDE, ppDDE, and opDDD in both maternal and infant hair at birth and at the ten-month follow-up. In particular, all these organochlorine compounds were detected at low but measurable concentrations in pregnant women, as well as in their newborns and infants at follow-up. Our results show that differences emerged between maternal and newborn levels of opDDE, opDDD, and ΣDDT. In newborns, our findings regarding DDT metabolites are consistent with effective placental transmission, as these lipid-soluble compounds can passively diffuse across biological membranes [77]. As a result, our findings provide convincing evidence that DDT metabolites are accumulated during infants’ early life.

This study has several limitations that should be acknowledged. First, the sample size was moderate, which may limit statistical power for subgroup analyses. This study’s small sample size in the ten-month follow-up group suggests that future studies with larger cohorts and extended follow-up are needed to confirm these EDC exposure patterns during infants’ early life and to better elucidate inter-infant variability. In line with this, the small number of twin pairs precluded meaningful twin-to-twin paired comparisons in the current study. Future studies with larger twin cohorts are warranted to fully leverage the potential of twins to assess EDC exposure in their early life. Second, hair exposure may be influenced by external environmental contamination [49], although rigorous washing protocols are applied to minimize this effect. In addition, the current study was biased in the information collected from pregnant women’s questionnaires, as it did not verify the accuracy of the data regarding product usage. Despite these limitations, the longitudinal design and paired mother–infant sampling provide strong evidence of dynamic early-life exposure patterns.

## 5. Conclusions

Through hair biomonitoring, this is the first longitudinal study to examine maternal and infant exposure to PBs, BPs, TCS, and organochlorine compounds. Our findings supplement existing urine biomonitoring data and provide strong, valuable evidence that hair is a unique substrate for assessing EDC exposure during birth and early life. In particular, our longitudinal design and paired mother–infant sampling enable analysis of changes in EDC exposure over time and the detection of postnatal accumulation patterns, thus offering new insights into the persistence of EDCs during critical developmental periods during infancy. In this way, the hair biomonitoring approach, which is rarely used in neonatal cohorts, proves to be a valuable tool for detecting chronic exposures over time. This study thus advances the scientific community’s understanding of human developmental toxicology and provides empirical evidence supporting the principles of the DOHaD hypothesis. To develop a complete exposure profile, future research should combine environmental and food sample analysis with multi-matrix biomonitoring (hair, urine, blood, and breast milk).

## Figures and Tables

**Table 1:** Validation parameters of the applied methodology for BPS, TCS, parabens, HCB, and DDT isomers.

	Rt (min)	Target Ion	Q1 Ion	Standards Linearity R^2^	Spiked Linearity R^2^	LOD (pg/mg)	LOQ (pg/mg)	% Recovery ±SD (*n* = 3)	% Accuracy ±SD (*n* = 3)	Precision (% RSD) (*n* = 3)
BPS	13.1	249.1	285.1	0.9973	0.9901	0.6	1.9	92.8 ± 13.3	114.2 ± 14.1	12.4
MeP	14.1	151.1	194.0	0.9965	0.9937	0.7	2.3	98.5 ± 7.0	84.5 ± 11.1	20.4
EthP	15.9	165.1	208.1	0.9958	0.9874	0.7	2.4	102.2 ± 20.1	117.6 ± 9.7	15.9
ProP	17.6	179.0	239.0	0.9979	0.9812	0.7	2.2	94.9 ± 19.8	115.2 ± 12.6	15.5
BenP	19.05	227.1	287.2	0.9980	0.9934	0.8	2.6	101.1 ± 22.1	113.7 ± 7.5	17.9
ButP	19.1	193.1	236.1	0.9952	0.9911	0.3	1.0	81.9 ± 18.3	122.4 ± 8.8	18.2
TCS	23.2	287.0	289.0	0.9980	0.9972	0.7	2.5	102.7 ± 18.3	84.5 ± 25.9	18.6
HCB	15.31	284.0	249.0	0.9842	0.9981	0.08	0.27	84.2 ± 14.1	103.0 ± 13.4	18.2
opDDE	22.40	246.0	318.0	0.9869	0.9952	0.06	0.21	73.8 ± 8.5	100.4 ± 10.7	16.0
ppDDE	23.75	246.0	318.0	0.9849	0.9924	0.08	0.26	72.6 ± 21.4	103.1 ± 14.2	17.4
opDDD	24.05	235.0	165.0	0.9808	0.9974	0.07	0.24	68.2 ± 26.1	97.2 ± 9.5	16.8

Rt: retention time, LOD: limit of detection, LOQ: limit of quantification, BPS: bisphenol S, MeP: methylparaben, EthP: ethylparaben, ProP: propylparaben, BenP: benzylparaben, ButP: butylparaben, TCS: triclosan, HCB: hexachlorobenzene, opDDE: op-dichlorodiphenyldichloroethylene, ppDDE: pp-dichlorodiphenyldichloroethylene, opDDD: op-dichlorodiphenyldichloroethane, RSD: relative standard deviation.

**Table 2:** Demographic data of mothers participating in the study per living area.

		Area					
		Rural		Urban		Total	
		Mean	SD	Mean	SD	Mean	SD
	Age (year)	30.7	3.7	34.1	4.8	33.1	4.7
	Height (cm)	165.50	4.26	165.83	6.07	165.73	5.54
	Weight before (kg)	66.5	11.8	69.1	14.8	68.3	13.9
	Weight at gestation week (kg)	76.7	12.0	78.9	12.2	78.2	12.1
	ΔWeight, gestational weight gain from pre-pregnancy to gestation week (kg)	10.25	5.35	9.76	8.06	9.91	7.29
	BMI before (kg/m^2^)	24.29	4.37	25.11	5.03	24.86	4.82
	ΒΜΙ at gestation week (kg)	28.06	4.61	28.71	4.35	28.52	4.40
	ΔBMI, Change in BMI from pre-pregnancy to gestation week (kg/m^2^)	3.77	1.99	3.60	2.92	3.65	2.65
	Gestation Week	35.03	3.46	34.7	2.4	34.8	2.8
		***n***	**%**	***n***	**%**	***n***	**%**
Education	Primary	0	0.0%	2	4.9%	2	3.4%
	Secondary	13	72.2%	14	34.1%	27	45.8%
	University	5	27.8%	25	61.0%	30	50.8%
Working Sector	Public	3	16.7%	6	14.6%	9	15.3%
	Private	8	44.4%	31	75.6%	39	66.1%
	Self-Employment	0	0.0%	2	4.9%	2	3.3%
	Unemployment	7	38.9%	2	4.9%	9	15.3%

BMI: body mass index, SD: standard deviation.

**Table 3:** Demographic data of cohort’s newborns in whole sample and per area of living area.

	Rural (21)		Urban (47)		Total (68)	
	Mean (SD)	Min–Max	Mean (SD)	Min–Max	Mean (SD)	Min–Max
Weight (g)	2336 (740)	850–3490	2273 (526)	900–3760	2292 (593)	850–3760
Height (cm)	46 (5)	35–52	46 (3)	35–54	46 (4)	35–54
HC (cm)	32 (3)	23–35	32 (2)	26–35	32 (2)	23–35
	***n***	**%**	***n***	**%**	***n***	**%**
Boy	14	66.7%	31	66%	45	66.2%
Girl	7	33.3%	16	33%	23	33.8%
Disorders						
Genital	0	0.0%	1	2.1%	1	1.5%
Respiratory	2	9.5%	10	21.3%	12	17.6%
Any	2	9.5%	11	23.4%	13	19.1%

HC: head circumference, SD: standard deviation.

**Table 4:** Self-reported frequency of personal-care product use before and during pregnancy (Ν(%)).

Product	Every Day Before	Every Day During	Weekly Before	Weekly During	Monthly Before	Monthly During	Never Before	Never During	x^2^p *
**Body deodorant**	47 (79.7)	34 (57.6)	9 (15.3)	18 (30.5)	0 (0.0)	2 (3.4)	3 (5.1)	5 (8.5)	<0.001
**Shower gel**	38 (64.4)	38 (64.4)	20 (33.9)	20 (33.9)	0 (0.0)	0 (0.0)	1 (1.7)	1 (1.7)	<0.001
**Shampoo**	25 (42.4)	22 (37.3)	34 (57.6)	37 (62.7)	0 (0.0)	0 (0.0)	0 (0.0)	0 (0.0)	0.453
**Perfume**	33 (55.9)	17 (28.8)	14 (23.7)	15 (25.4)	6 (10.2)	9 (15.3)	6 (10.2)	18 (30.5)	<0.001
**Make-up**	28 (47.5)	14 (23.7)	14 (23.7)	16 (27.1)	15 (25.4)	20 (33.9)	2 (3.4)	9 (15.3)	0.007
**Nail polish**	4 (6.8)	1 (1.7)	11 (18.6)	1 (1.7)	30 (50.8)	22 (37.3)	14 (23.7)	35 (59.3)	<0.001
**Hair spray/gel**	6 (10.2)	2 (3.4)	17 (28.8)	10 (16.9)	13 (22.0)	9 (15.3)	23 (39.0)	38 (64.4)	0.004
**Body cream/lotion**	13 (22.0)	7 (11.9)	28 (47.5)	21 (35.6)	6 (10.2)	8 (13.6)	12 (20.3)	23 (39.0)	0.003
**Sunscreen**	14 (23.7)	9 (15.3)	17 (28.8)	11 (18.6)	12 (20.3)	10 (16.9)	16 (27.1)	29 (49.2)	0.019
**Hair dye**	1 (1.7)	0 (0.0)	0 (0.0)	0 (0.0)	39 (66.1)	9 (15.3)	19 (32.2)	50 (84.7)	0.066

* Significance assessed with the McNemar–Bowker test of symmetry.

**Table 5:** The percent frequencies of detection and mean (mean ± SD; min–max) concentration levels were measured (in pg/mg) in hair samples of mothers and infants at birth and at follow-up.

Analyte	Mothers	Infants at Birth	Infants at Follow-Up
**BPS**	100.0% (75.42 ± 83.64; 12.32–362.21)	87.1% (79.24 ± 251.21; 0.0–1930.62)	87.5% (289.2 ± 250.98; 0.0–960.44)
**MeP**	100.0% (4184.16 ± 6565.91; 24.19–28811.82)	100.0% (1035.56 ± 1465.96; 17.15–8777.84)	95.8% (1412.87 ± 2219.13; 0.0–7956.63)
**EthP**	92.6% (335.93 ± 539.63; 0.0–2090.94)	38.7% (25.54 ± 39.87; 0.0–162.39)	70.8% (85.88 ± 108.2; 0.0–468.1)
**ProP**	100.0% (691.99 ± 1622.63; 17.9–7939.76)	95.2% (460.62 ± 589.22; 0.0–3070.01)	83.3% (1003.41 ± 2292.79; 0.0–10182.51)
**BenP**	61.1% (37.82 ± 64.76; 0.0–292.78)	25.8% (8.45 ± 19.64; 0.0–126.18)	29.2% (19.39 ± 39.86; 0.0–152.98)
**ButP**	50.0% (38.31 ± 86.54; 0.0–568.64)	17.7% (6.64 ± 19.92; 0.0–137.67)	37.5% (21.19 ± 46.24; 0.0–191.93)
**TCS**	100.0% (1303.09 ± 1529.56; 19.43–5619.14)	100.0% (2840.14 ± 2302.38; 160.27–9435.74)	87.0% (881.47 ± 2251.94; 0.0–8450.6)
**HCB**	76.0% (5.06 ± 5.96; 0.0–23.33)	75.0% (5.58 ± 5.79; 0.0–28.52)	63.6% (3.04 ± 3.75; 0.0–11.43)
**opDDE**	44.0% (0.43 ± 0.53; 0.0–1.94)	73.3% (1.95 ± 5.32; 0.0–34.79)	72.7% (3.44 ± 5.12; 0.0–19.53)
**ppDDE**	90.0% (3.07 ± 3.03; 0.0–12.33)	88.3% (8.71 ± 23.71; 0.0–160.66)	77.3% (6.37 ± 9.79; 0.0–35.62)
**opDDD**	8.0% (0.04 ± 0.14; 0.0–0.71)	31.7% (0.71 ± 3.08; 0.0–23.62)	18.2% (2.46 ± 8.12; 0.0–34.73)
**SumDDTs**	90.0% (3.53 ± 3.2; 0.0–14.27)	91.8% (11.98 ± 33.92; 0.0–225.65)	95.5% (12.34 ± 19.66; 0.0–82.53)

BPS: bisphenol S, MeP: methylparaben, EthP: ethylparaben, ProP: propylparaben, BenP: benzylparaben, ButP: butylparaben, TCS: triclosan, HCB: hexachlorobenzene, opDDE: op-dichlorodiphenyldichloroethylene, ppDDE: pp-dichlorodiphenyldichloroethylene, opDDD: op-dichlorodiphenyldichloroethane.

**Table 6:** Concentration levels (median, IQR) were measured (in pg/mg) in hair of infants by feeding type (breastfed, mixed-fed).

Analyte	Breastfed	Mixed	*p*-Value *
**BPS**	218.73 (130.17–594.16)	276.98 (150.56–319.51)	0.841
**MeP**	953.15 (119.11–4812.35)	414.67 (229.73–1347.93)	0.920
**EthP**	0.00 (0.00–30.74)	68.60 (35.36–116.55)	0.081
**ProP**	157.37 (21.16–759.75)	280.84 (87.12–799.70)	0.651
**BenP**	0.00 (0.00–35.40)	0.00 (0.00–27.82)	0.686
**ButP**	10.27 (0.00–28.10)	0.00 (0.00–20.58)	0.729
**TCS**	154.66 (39.34–388.89)	100.61 (81.56–456.87)	0.457
**HCB**	0.45 (0.00–8.51)	1.87 (0.00–5.73)	0.702
**opDDE**	1.67 (1.25–2.24)	1.00 (0.00–5.19)	0.612
**ppDDE**	0.00 (0.00–4.40)	4.28 (1.57–7.48)	0.251
**SumDDT**	2.24 (1.25–6.07)	5.33 (4.41–12.67)	0.257

BPS: bisphenol S, MeP: methylparaben, EthP: ethylparaben, ProP: propylparaben, BenP: benzylparaben, ButP: butylparaben, TCS: triclosan, HCB: hexachlorobenzene opDDE: op-dichlorodiphenyldichloroethylene, ppDDE: pp-dichlorodiphenyldichloroethylene, opDDD: op-dichlorodiphenyldichloroethane; * significance assessed with the Mann–Whitney test.

**Table 7:** Concentration (median, IQR) levels were measured (in pg/mg) of each analyte in hair of infants at birth and at follow-up by sex.

	At Birth			At Follow-Up		
Analyte	Boys	Girls	*p*-Value *	Boys	Girls	*p*-Value *
**BPS**	18.78 (13.25–44.47)	23.86 (16.90–47.92)	0.252	282.69 (149.44–616.46)	227.88 (84.65–271.28)	0.290
**MeP**	584.04 (165.62–1099.20)	451.74 (268.75–1344.53)	0.821	505.93 (238.22–2614.89)	288.81 (94.60–949.39)	0.215
**EthP**	0.00 (0.00–39.22)	0.00 (0.00–66.69)	0.560	65.07 (0.00–152.81)	35.36 (0.00–72.12)	0.290
**ProP**	206.84 (105.13–558.97)	175.22 (126.24–510.43)	0.976	272.43 (42.31–1233.81)	145.06 (61.14–540.38)	0.640
**BenP**	0.00 (0.00–0.00)	0.00 (0.00–16.66)	0.681	0.00 (0.00–0.00)	0.00 (0.00–27.82)	0.519
**ButP**	0.00 (0.00–0.00)	0.00 (0.00–10.35)	0.298	0.00 (0.00–20.54)	20.58 (0.00–26.28)	0.263
**TCS**	2158.79 (1242.35–4022.91)	2086.61 (1384.41–3510.68)	0.940	92.08 (54.61–456.87)	181.03 (32.93–483.75)	0.875
**HCB**	4.41 (1.91–6.99)	3.28 (0.00–9.85)	0.752	2.49 (0.00–7.18)	0.42 (0.00–2.92)	0.238
**opDDE**	0.88 (0.30–1.62)	0.95 (0.00–1.83)	0.764	1.71 (0.00–4.41)	0.91 (0.72–5.62)	0.815
**ppDDE**	2.72 (1.29–4.56)	3.12 (1.33–5.14)	0.695	3.76 (1.54–7.48)	2.41 (0.58–4.58)	0.402
**opDDD**	0.00 (0.00–0.36)	0.00 (0.00–1.03)	0.154	0.00 (0.00–0.77)	0.00 (0.00–0.00)	0.297
**SumDDΤ**	3.58 (2.26–8.53)	4.44 (2.13–10.14)	0.481	5.33 (3.12–12.67)	4.52 (2.07–7.91)	0.365

BPS: bisphenol S, MeP: methylparaben, EthP: ethylparaben, ProP: propylparaben, BenP: benzylparaben, ButP: butylparaben, TCS: triclosan, HCB: hexachlorobenzene, opDDE: op-dichlorodiphenyldichloroethylene, ppDDE: pp-dichlorodiphenyldichloroethylene, opDDD: op-dichlorodiphenyldichloroethane; * significance assessed with the Mann–Whitney test.

**Table 8:** Pairwise comparisons of analyte concentrations between mothers and infants at birth and at follow-up.

Analyte	Group	N	Mean (SD) (pg/mg)	Median (IQR) (pg/mg)	Wilcoxon Signed Ranks Test—*p* Values (Pair)
**BPS**	Mother	54	75.42 (83.64)	51.08 (22.76–89.65)	0.001 */<0.0001 **/0.001 ***
	Infant at birth	62	79.24 (251.21)	19.35 (13.98–46.64)	
	Infant at follow-up	24	289.20 (250.98)	250.40 (100.85–319.09)	
**MeP**	Mother	54	4184.16 (6565.91)	1151.80 (214.56–7066.58)	0.006/0.065/0.075
	Infant at birth	62	1035.56 (1465.96)	487.63 (184.85–1180.18)	
	Infant at follow-up	24	1412.87 (2219.13)	414.67 (146.31–1588.05)	
**EthP**	Mother	54	335.93 (539.63)	79.51 (45.80–435.60)	<0.0001/0.001/0.122
	Infant at birth	62	25.54 (39.87)	0.00 (0.00–44.34)	
	Infant at follow-up	24	85.88 (108.20)	62.10 (0.00–116.53)	
**ProP**	Mother	54	691.99 (1622.63)	188.04 (51.65–570.52)	0.160/0.726/0.151
	Infant at birth	62	460.62 (589.22)	195.23 (111.72–570.22)	
	Infant at follow-up	24	1003.41 (2292.79)	175.22 (47.02–747.77)	
**BenP**	Mother	54	37.82 (64.76)	19.15 (0.00–40.07)	0.001/0.070/0.594
	Infant at birth	62	8.45 (19.64)	0.00 (0.00–15.44)	
	Infant at follow-up	24	19.39 (39.86)	0.00 (0.00–23.23)	
**ButP**	Mother	54	38.31 (86.54)	9.79 (0.00–36.86)	<0.0001/0.070/0.424
	Infant at birth	62	6.64 (19.92)	0.00 (0.00–0.00)	
	Infant at follow-up	24	21.19 (46.24)	0.00 (0.00–24.41)	
**TCS**	Mother	54	1303.09 (1529.56)	595.12 (211.46–2011.26)	<0.0001/0.014/0.001
	Infant at birth	62	2840.14 (2302.38)	2115.46 (1252.67–3777.59)	
	Infant at follow-up	23	881.47 (2251.94)	97.15 (54.61–456.87)	
**HCB**	Mother	50	5.06 (5.96)	3.09 (0.55–7.16)	0.168/0.179/0.904
	Infant at birth	60	5.58 (5.79)	4.13 (0.11–7.95)	
	Infant at follow-up	22	3.04 (3.75)	1.13 (0.00–5.76)	
**opDDE**	Mother	50	0.43 (0.53)	0.00 (0.00–0.84)	0.007/0.003/0.079
	Infant at birth	60	1.95 (5.32)	0.90 (0.00–1.64)	
	Infant at follow-up	22	3.44 (5.12)	1.13 (0.00–4.60)	
**ppDDE**	Mother	50	3.07 (3.03)	2.26 (1.23–4.05)	0.147/0.232/0.874
	Infant at birth	60	8.71 (23.71)	2.88 (1.31–4.90)	
	Infant at follow-up	22	6.37 (9.79)	3.21 (0.87–6.33)	
**opDDD**	Mother	50	0.04 (0.14)	0.00 (0.00–0.00)	0.002/0.080/0.953
	Infant at birth	60	0.71 (3.08)	0.00 (0.00–0.42)	
	Infant at follow-up	22	2.46 (8.12)	0.00 (0.00–0.00)	
**ΣDDT**	Mother	50	3.53 (3.20)	3.04 (1.50–4.45)	0.008/0.006/0.702
	Infant at birth	49	11.98 (33.92)	3.81 (2.19–8.57)	
	Infant at follow-up	22	12.34 (19.66)	5.06 (2.24–12.19)	

SD: standard deviation, IQR: interquartile range, picogram/milligram (pg/mg), BPS: bisphenol S, MeP: methylparaben, EthP: ethylparaben, ProP: propylparaben, BenP: benzylparaben, ButP: butylparaben, TCS: triclosan, HCB: hexachlorobenzene, opDDE: op-dichlorodiphenyldichloroethylene, ppDDE: pp-dichlorodiphenyldichloroethylene, opDDD: op-dichlorodiphenyldichloroethane;* mother vs. infant at birth, ** mother vs. infant at follow-up, *** infant at birth vs. infant at follow-up.

## Data Availability

The original contributions presented in this study are included in the article. Further inquiries can be directed to the corresponding authors.
